# Cross-union surgery for tibiofibular fractures in McCune-Albright syndrome: a case report and literature review

**DOI:** 10.3389/fped.2025.1663626

**Published:** 2025-10-14

**Authors:** Hui Zhu, Yiwei Wang, Hanjie Zhuang, Pengfei Zheng, Yuping Tang

**Affiliations:** Department of Orthopaedic Surgery, Children’s Hospital of Nanjing Medical University, Nanjing, Jiangsu, China

**Keywords:** pseudarthrosis, tibial fractures, fibrous dysplasia, polyostotic, cross-union, congenital pseudoarthrosis of tibia

## Abstract

**Introduction:**

McCune-Albright syndrome (MAS) is a rare disease. MAS manifests in the skeletal system as Fibrous dysplasia (FD). In MAS patients, bone lesions often result in fractures under weight-bearing forces during childhood, and pseudarthrosis formation is highly likely after fractures. There is currently no ideal treatment for fractures in children with MAS. This case study presents the innovative application of the cross-union technique in treating a 14-year-old female MAS patient with a distal tibial and fibular fracture nonunion.

**Methods:**

The patient had a history of conservative treatment with persistent symptoms, including an inability to bear weight and walk independently. A “cross-union” procedure was performed using a Fassier-Duval rod, locking plate, and K-wire fixation.

**Results:**

At the final follow-up 12 months postoperatively, x-rays revealed a stable “cross-union” bone connection at the distal ends of the tibia and fibula, with no occurrence of refracture or other complications. The Radiographic Union Score for Tibial Fractures (RUST) was 12, and the Olerud Molander Ankle Score was 60.

**Conclusion:**

We successfully applied the “cross-union” technique to a MAS child with nonunion after a distal tibiofibular fracture. We look forward to further improvement and promotion of the “cross-union” technology in the future, bringing new hope for treating fractures in MAS/FD children.

## Introduction

McCune-Albright syndrome (MAS) is a rare disease, with an incidence of approximately 1/100,000–1/1,000,000 (1–4). The original description of MAS included a “classic triad” of café-au-lait macules, precocious puberty, and Fibrous dysplasia (FD) (3). MAS manifests in the skeletal system as FD (5). Involvement of long bones can lead to pain, with elongated and irregular bony trabeculae making the bone brittle. In MAS patients, bone lesions often result in fractures under weight-bearing forces during childhood (6), and pseudarthrosis formation is highly likely after fractures. Surgical intervention is usually considered for children with fractures. However, postoperative complications such as nonunion, malunion, refracture, and loosening of internal fixation remain high (2), posing a significant clinical challenge. Therefore, there is currently no ideal treatment for fractures in children with MAS.

In 2011, Choi et al. (7) proposed a new method called “4-in-1 osteosynthesis” for the treatment of Congenital pseudarthrosis of the tibia. In 2012, Paley et al. (8) utilized a similar “cross-union” method for the treatment of Congenital Pseudarthrosis of the Tibia (CPT). Both studies demonstrated that stable osseous unions were achieved at nonunion sites after “cross-union” surgery, significantly reducing the refracture rate. In this case, we report on a child with McCune-Albright syndrome (MAS) who developed nonunion after a distal tibiofibular fracture. Considering that nonunion in MAS patients is very similar to congenital pseudarthrosis of the tibia, we innovatively used the “cross-union” technique for treatment.

## Methods

**Figure 1:**
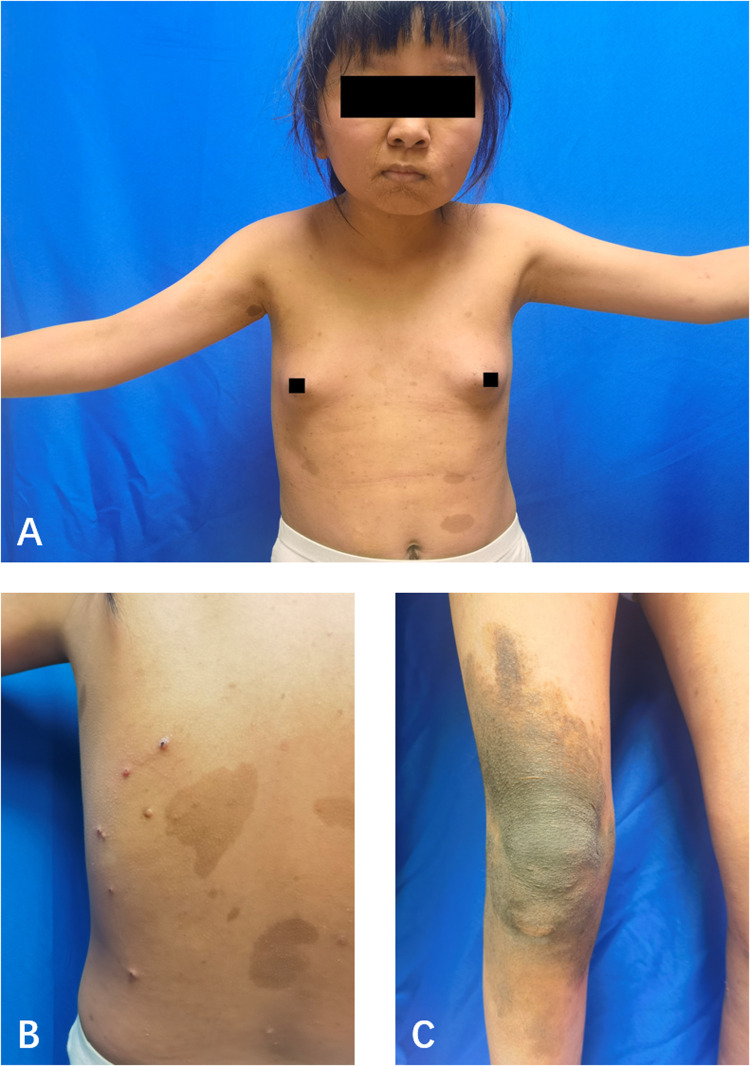
The appearance of the child. **(A)** The front of the upper body. **(B)** A part of the back. **(C)** A part of the right knee joint. Breast development and café-au-lait macules.

**Figure 2:**
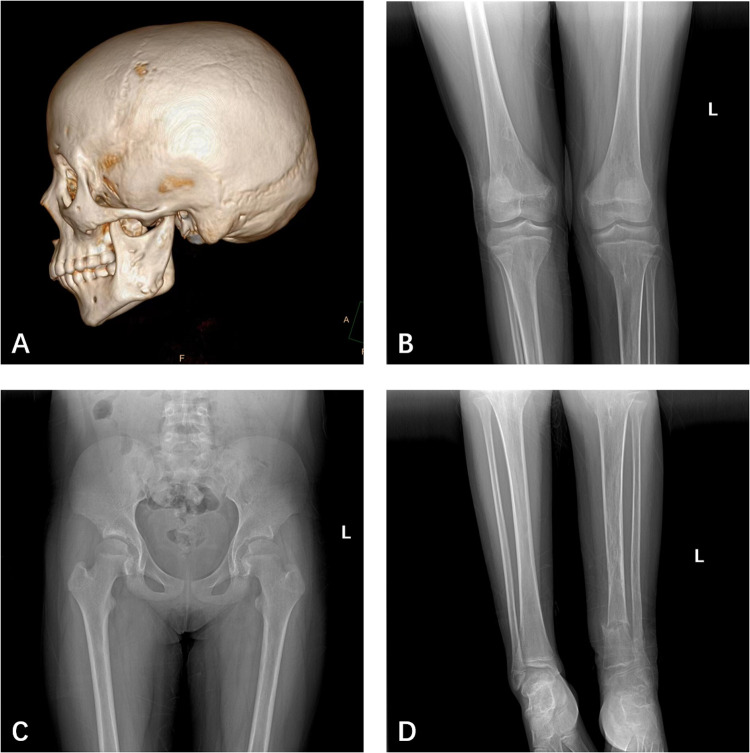
**(A)** CT showed bone abnormalities in the left parietal bone, and left mandible. **(B)** X-ray showed bone abnormalities in both femurs, tibia, and fibula. **(C)** X-ray showed bone abnormalities in both femurs, but not the iliac bone. **(D)** X-ray showed the left distal tibia and fibula fractures and nonunion.

**Figure 3:**
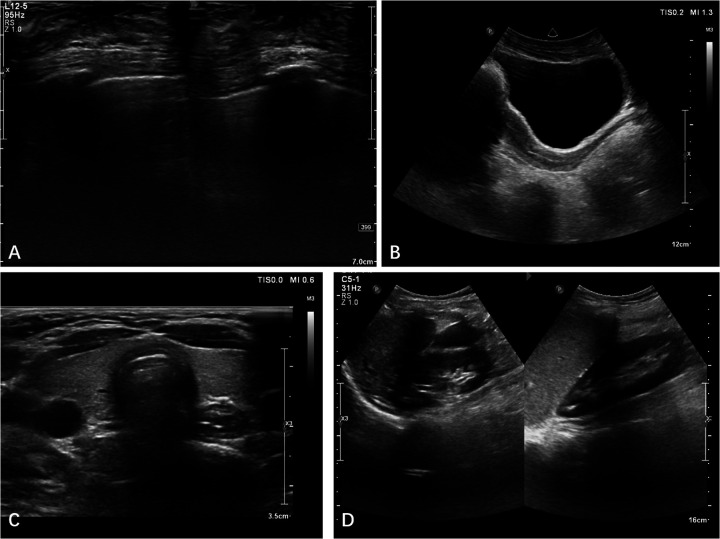
Ultrasound revealed bilateral breast enlargement **(A)** and enlarged uterus **(B)**, while thyroid **(C)** and adrenal glands **(D)** showed no abnormalities.

The patient, a 14-year-old female, experienced a fall while running. Initially, she complained of left leg and ankle pain and was unable to bear weight. However, she did not seek medical attention and continued with the weight-bearing activities, and her parents didn't notice the abnormality. After three months with persistent symptoms, she presented to a medical institution where an x-ray revealed a distal tibiofibular fracture. She underwent two months of cast immobilization followed by one month of external fixator fixation. No specify drug was intaken during this period. Follow-up x-rays showed nonunion at the fracture ends. Then she visited our outpatient clinic. Physical examination revealed facial asymmetry, multiple café-au-lait spots over her body, breast development, approximately 10° external rotation deformity of the distal left leg, limited active movement of the left ankle with pain on passive movement, and inability to bear weight independently (Figure 1). Comprehensive x-rays and CT scans identified nonunion of the left distal tibiofibular fracture in addition to the abnormal bone structure in the bilateral femurs, tibiae, left parietal bone and left mandible (Figure 2). Ultrasound revealed bilateral breast enlargement (Figure 3A) and enlarged uterus (Figure 3B), while thyroid (Figure 3C) and adrenal glands (Figure 3D) showed no abnormalities. Hematological hormone tests were normal (Table 1), as were whole exome sequencing tests, which showed negative results. Based on clinical examination and laboratory findings, she was diagnosed with MAS.

**Table 1:** Hematological hormone tests.

Hormone	Results
Insulin-like growth factor 1	265 ng/ml
Ultrasensitive growth hormone	0.088 ng/ml
Hydrocortisone	418.6 nmol/L
Adrenocorticotropic hormone	37.77 pg/ml
Insulin	8.99 mU/L
C-peptide	0.488 nmol/L
Triiodothyronine	2.75 nmol/L
Free triiodothyronine	6.17 pmol/L
Thyroxine	97.32 nmol/L
Free thyroxine	16.79 pmol/L
Thyroid stimulating hormone	2.9 uIU/ml
Estradiol	142.7 pmol/L
Progesterone	1.69 nmol/L
Testosterone	<0.087 nmol/L
Prolactin	101 mIU/L
Follicle stimulating hormone	8.39 mIU/ml
Luteinizing hormone	6.74 mIU/ml

When the guardians of this patient opted for surgery, the pseudarthrosis had been present for an extended period and had resulted in secondary ankle valgus deformity. Considering the complexity of the child's condition, we were concerned that addressing both the nonunion and the ankle valgus deformity simultaneously in a single procedure might lead to suboptimal postoperative recovery due to the inherent nature of the fracture, potentially compromising the overall outcome. Therefore, a two-stage surgical plan was devised: the first stage involved “cross-union” surgery of the distal tibia and fibula, with a second stage planned for correction of angular deformity.

**Figure 4:**
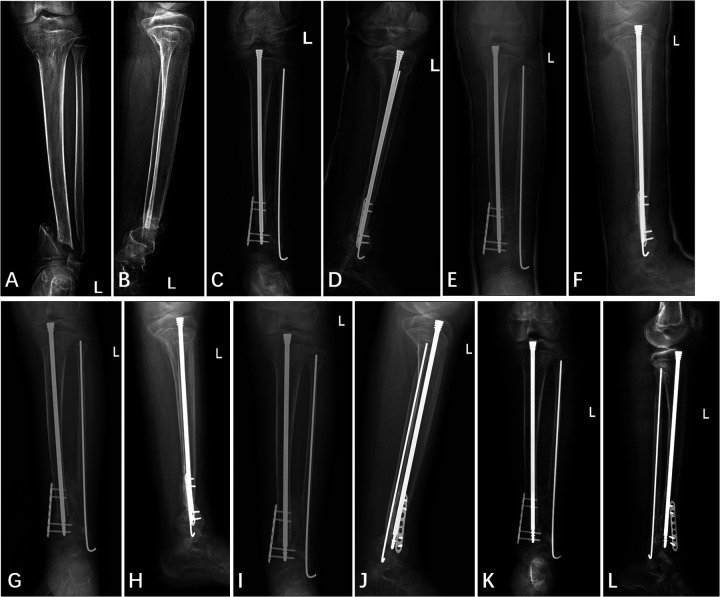
Anterior and lateral x-ray film of left tibia and fibula **(A,B)** one day pre-operation, **(C,D)** one day post-operation, **(E,F)** two months post-operation, **(G,H)** four months post-operation, **(I,J)** five months post-operation, and **(K,L)** twelve months post-operation.

The patient underwent the first stage of “cross-union” surgery at our institution. Based on preoperative imaging localization (Figures 4A,B), a longitudinal incision approximately 10 cm in length was made over the anterior aspect of the left lower leg at the site of the nonunion. Anterior and deep posterior fasciotomy and muscle reflection to expose tibia, interosseous membrane, and fibula, allowed procedures to be performed under direct vision without damage to the neurovascular bundles. Fracture ends were separated, fibrous connections and sclerotic bone were removed, over the planned length of the cross-union. The medullary cavity of the tibia and fibula was penetrated to prepare for internal fixation implantation. During the preoperative physical examination, we identified an Achilles tendon contracture on the left side, which was further confirmed during the surgery. Therefore, a second incision was made posteriorly over the distal left lower leg to perform percutaneous lengthening of the left Achilles tendon. Subsequently, a customized Fassier-Duval telescopic nail is inserted to maintain tibial length and accommodate future bone growth, the male end locked into the distal epiphysis and the female end screwed into the proximal epiphysis. A seven-hole locking plate and four screws were used to fix the distal tibia to achieve enhanced local stability and prevent rotation. A 2.5 mm Kirschner wire was used for intramedullary fixation of the fibula to further reinforce local stability. A third incision, approximately 8 cm long, was made over the iliac crest. A periosteal graft is harvested from the undersurface of the iliacus muscle. It is then expanded by passing it through the skin graft mesher. Decancellousization of the ilium is done by first splitting the two cortical tables of the ilium down to the roof of the acetabulum, triradiate cartilage, sciatic notch, posterior spines and sacro-iliac joint. The periosteal graft is wrapped around the fracture ends and bone morphogenic protein-2 (BMP2) collagen sponges are inserted overtop the posterior muscles behind the tibia and fibula. The cancellous bone is inserted between the tibia and fibula. Another BMP2 sponges are placed overtop the bone graft. The anterior muscles lie over the BMP2, ultimately forming a five-layer sandwich structure of muscle–BMP2–graft–BMP2–muscle (8, 9). Considering the potential for significant postoperative bleeding, a long-leg posterior splint was applied with a drainage tube in place immediately following surgery (Figures 4C,D). The drain was removed 7 days postoperatively, at which point the immobilization was converted to a long-leg cylindrical cast. The patient was followed up monthly with radiographic examination. During the casting period, weight bearing on the affected limb was strictly prohibited. No specify drug other than antibiotic was used.

The study was conducted in accordance with the Declaration of Helsinki, and approved by the Institutional Review Board of Children's Hospital of Nanjing Medical University (No. 9712).

## Results

Callus began to appear approximately two months after surgery (Figures 4E,F). The decision to remove the cast at four months was based primarily on the radiographic evidence of healing, specifically, the observation of continuous callus formation bridging the non-union site (Figures 4G,H). The patient was allowed to start protected weight bearing with crutches and an orthosis after cast removal, and progressed to independent weight bearing without assistance by approximately 5 months postoperatively. At the final follow-up 12 months postoperatively, x-rays revealed a stable “cross-union” bone connection at the distal ends of the tibia and fibula, with no occurrence of refracture or other complications (Figures 4I,J). The Radiographic Union Score for Tibial fractures (RUST) was calculated using standard anterior-posterior and lateral views (Table 2). At the two-year postoperative mark, the patient did not return to our institution for radiographic follow-up. However, a telephone survey was conducted to assess the recovery of her lower extremity motor function. Functional recovery assessment was performed using the Olerud Molander Ankle Score (OMAS) (Table 3).

**Table 2:** Radiographic union score for tibial fractures (RUST) of the patient at the final follow-up 12 months postoperatively.

Cortex	Score	Total score
Anterior	3 (No Fracture Line, Bridging Callus)	12
Posterior	3 (No Fracture Line, Bridging Callus)	
Lateral	3 (No Fracture Line, Bridging Callus)	
Medial	3 (No Fracture Line, Bridging Callus)	

**Table 3:** Olerud molander ankle score (OMAS) of the patient at the final follow-up 12 months postoperatively.

Parameter	Degree	Score	Total score
Pain	None	25	60
Stiffness	Stiffness	0	
Swelling	None	10	
Stair-climbing	Impaired	5	
Running	Impossible	0	
Jumping	Impossible	0	
Squatting	Impossible	0	
Supports	None	10	
Work, activities of daily life	Change to a simpler work	10	

## Discussion

Children with MAS are prone to fractures and nonunion after fractures, and there is currently no ideal treatment for fractures in MAS children. In this case, we report a child with MAS who developed pseudarthrosis following a distal tibiofibular fracture, and was successfully treated using the “cross-union” technique.

MAS results from a chimeric mutation in the GNAS gene located on chromosome 20q13.3 occurring during early embryogenesis (10). Due to the mutation being distributed in a mosaic pattern, MAS can affect multiple systems with a highly variable presentation and random combination of symptoms (3). MAS manifests in the skeletal system as FD, where the gene mutation leads to an increase in intracellular cAMP signaling, causing osteoblasts to differentiate into stromal cells while inhibiting further differentiation (5).Currently, the mainstay of treatment for fractures in MAS/FD patients is surgery, focusing on maintaining mechanical stability and preventing complications such as malunion (3). After a thorough examination, clinicians tailor the treatment plan based on the characteristics of the fracture (11). For tibiofibular fractures, conservative treatment can be initially considered, but surgical intervention is warranted in cases of repeated fractures, malunion, or functional impairment (3). In this case, the patient had a history of conservative treatment, radiological evidence of nonunion, and an inability to bear weight or walk independently, so conservative treatment was not considered. Biomechanical factors make fractures most common in the proximal femur in MAS/FD patients (3), with distal tibiofibular fractures being rarer. There is limited literature on the treatment of fractures in locations other than the proximal femur. While some studies have explored surgical treatment of fractures in MAS/FD patients, no consensus exists on the best approach. Fang et al. (11) conducted a retrospective study on 22 FD patients who underwent surgery for fractures, but only four cases involved tibial fractures, each treated with different surgical techniques, and follow-up showed no related complications with satisfactory imaging results. In terms of surgical technique, intramedullary nails are typically preferred over plates and screws due to the lack of normal bone for fixation (12). Ippolito et al. (13) conducted a long-term follow-up study on the use of intramedullary nails in FD patients, including eight with fractures, and found an 81% satisfaction rate with long-term outcomes, though the complication rate remained high at 32.5%. Another study by Ippolito et al. (14) examined the use of intramedullary nails in FD patients with previous unsuccessful lower limb treatments, showing improved lower limb function in all patients, but a 21% complication rate. Additionally, the healing time for FD fractures is typically longer, and if a patient's bones grow rapidly during intramedullary nail fixation, removal or replacement of the nail may be necessary to avoid impacting treatment efficacy (13). Therefore, intramedullary nails are an effective treatment for FD patients but have a high complication rate.

In 1963, Bailey and Dubow developed the Bailey-Dubow extendable intramedullary nail for treating osteogenesis imperfecta (15). This was followed by the development of Fassier-Duval telescopic rods and Interlocking Telescopic Rods, which have the advantage of extending with the growth of long bones compared to standard intramedullary nails. Spahn et al. (16) compared the success rates of Fassier-Duval telescopic rods with static fixation devices, finding that Fassier-Duval rods significantly reduced the need for repeat surgeries in immature limbs. Shin et al. (17) developed a Dual Interlocking Telescopic Rod and studied its effectiveness in treating tibial fractures in osteogenesis imperfecta patients, demonstrating effective stabilization of the tibia. Extendable intramedullary nails are also used in treating congenital pseudarthrosis of the tibia (CPT) and other bone disorders (16, 17).

CPT is a rare condition. When presenting without tibial fracture, the standard of care is to prevent fractures through bracing (9, 18). The four main treatments for CPT include intramedullary nail fixation, Ilizarov external fixation, and vascularized fibular grafting, but high rates of nonunion and refracture remain (9, 18–20). Choi et al. (7) and Paley et al. (8) independently proposed the “cross-union” technique for CPT treatment in 2011 and 2012, respectively. Paley et al. detailed the “cross-union” procedure (8), showing satisfactory outcomes with 100% osseous union and a 0% refracture rate.

Inspired by these findings and considering the similar pathophysiology of nonunion in MAS fractures and CPT, we innovatively applied the “cross-union” technique to a case of distal tibiofibular fracture nonunion in a MAS patient. Although genetic testing for this patient was negative, the mosaic pattern of mutations in MAS warranted the diagnosis based on clinical examination and testing. During the “cross-union” surgery, a cancellous bone from the iliac crest was used, and unlike CPT patients, MAS patients may also have iliac involvement. Therefore, comprehensive pelvic imaging was performed preoperatively, and normal bone quality was confirmed intraoperatively. Ideally, angular deformities should be completely corrected during surgery, but due to the prolonged nonunion period and significant angular deformity at the fracture ends, immediate correction might have compromised stability and the success of the “cross-union”. Thus, partial angular deformity was retained.

At the 12-month postoperative follow-up, the patient showed a “cross-union” at the fracture site with no refracture. A RUST (21) of 12 signifies complete healing as evidenced by the absence of fracture lines and the presence of bridging callus across all four cortices. This scoring reflects advanced stages of bone healing and suggests that the patient has achieved satisfactory radiographic union. The OMAS (22) results indicate varying degrees of functional recovery in different aspects: pain and swelling scores are high, while mobility and activity scores are lower, suggesting ongoing limitations in physical activities due to the residual angular deformity at the distal tibia and fibula. These findings complement the RUST score. Currently, we plan to perform a secondary surgery to correct the angular deformity in the future.

Using the “cross-union” technique to treat children with MAS/FD accompanied by fractures, especially non-unions, offers significant advantages compared to other traditional methods. As mentioned previously, there is currently no ideal treatment for nonunion in children with MAS/FD. The most common approaches remain curettage and bone grafting with or without internal fixation and osteotomy (11–13). The grafts used are typically massive allografts, and internal fixation usually consists of intramedullary nails or plate and screws. These treatments still carry risks of complications such as nonunion and refracture after healing. Due to the rarity of such cases, few studies have provided detailed statistical analysis of the incidence of these complications. In comparison to conventional methods, the cross-union technique employs autologous cancellous bone grafting, which offers better biocompatibility than allografts. Additionally, cancellous bone has superior osteogenic efficiency compared to cortical bone (8). In terms of implant choice, the use of intramedullary nails or plates carries a 67% probability of requiring revision surgery in the future (13). The cross-union technique significantly reduces the risk of implant failure and the need for revision by combining a Fassier-Duval rod with a plate. Finally, the greatest strength of the cross-union technique lies in its formation of a “4-in-1” structure, preventing non-union and re-fracture, thereby improving treatment outcomes.

At the two-year postoperative mark, the patient has not experienced any complications to date. Nevertheless, long-term monitoring remains necessary due to the potential for future complications such as refracture or hardware failure. Although failure of cross-union formation represents one of the most serious potential complications of this technique, it has not been reported in previous studies employing cross-union for the treatment of CPT (7–9, 23). Furthermore, the patient presented with ankle deformity and joint dysfunction, which cannot be directly resolved by cross-union surgery. There remains a risk of symptom progression or other related complications in the future. However, the patient's guardians have currently adopted a conservative approach toward secondary corrective surgery. Overall, while the application of cross-union carries certain long-term risks, both published outcomes and our follow-up observations to date suggest that the likelihood of these complications is relatively low.

This study has several limitations. As a single case report with only 12 months of follow-up, the long-term efficacy and potential complications of “cross-union” in MAS remain uncertain. This treatment still needs more trial and validation before clinical application. Additionally, the delay in the treatment of this child resulted in the need for staged surgery, which increases the physical, mental and economic burden and may affect the final treatment effect to a certain extent.

## Conclusion

We successfully applied the “cross-union” technique to a MAS child with nonunion after a distal tibiofibular fracture. We look forward to further improvement and promotion of the “cross-union” technology in the future, bringing new hope for treating nonunion after fractures in MAS/FD children.

## Data Availability

The datasets generated during the current study are available from the corresponding author on reasonable request.
